# Role of TaqMan array card in determining causative organisms of acute febrile illness in hospitalized patients

**DOI:** 10.1002/jcla.24948

**Published:** 2023-07-27

**Authors:** Tabassum Ferdousi, Asok Kumar Dutta, M. A. Hassan Chowdhury, Kamrul Islam, Md. Taufiqul Islam, Md. Zahirul Islam, Md. Rakibul Hassan Bulbul, Ashraful Islam Khan, Firdausi Qadri

**Affiliations:** ^1^ Bangladesh Institute of Tropical and Infectious Diseases Chattogram Bangladesh; ^2^ Chattogram Medical College Hospital Chattogram Bangladesh; ^3^ International Centre for Diarrheal Disease Research, Bangladesh (icddr,b) Dhaka Bangladesh; ^4^ Institute for Developing Science and Health Initiatives (ideSHi) Dhaka Bangladesh

## Abstract

**Background:**

Acute febrile illness (AFI) is a prevalent disease in developing countries that is difficult to diagnose due to the diversity of infectious organisms and the poor quality of clinical diagnosis. TaqMan array card (TAC) can detect up to 35 AFI‐associated organisms in 1.5 h, addressing diagnostic demands. In this study, we aimed to evaluate the role of TAC in determining the causative organisms in hospitalized AFI patients.

**Methods:**

The study had a cross‐sectional design and enrolled 120 admitted patients with persistent fever for three or more days from the medicine ward of Chittagong Medical College Hospital (CMCH) and Bangladesh Institute of Tropical and Infectious Diseases Hospital (BITID). Blood samples were collected and then subjected to automated BacT/Alert blood culture, microbial culture, TAC assay, and typhoid/paratyphoid test.

**Results:**

The total number of study participants was 120, among them 48 (40%) samples showed a positive result in TAC card, 29 (24.16%) were TP positive and nine (7.51%) were culture positive. The number of organisms detected by the TAC card was 13 bacteria, three viruses, one protozoan, and one fungus. The sensitivity and specificity of the TAC assay for different bacterial pathogen compared to blood culture was 44.44%, and 90.99%, respectively. In contrast, the TP test had a sensitivity and specificity of 100% and 80%, respectively, compared to the blood culture test.

**Conclusion:**

TAC can be a handful tool for detecting multiple organisms in AFI with high specificity which can facilitate early diagnosis of different pathogens contributing to AFI.

## Graphical abstract

TaqMan array card (TAC); a rapid, multiplex RT‐PCR‐based molecular assay, can facilitate up to seven clinical samples simultaneously within 1.5 h. The aim of this study was to evaluate the role of TAC in determining the causative organism in hospitalized AFI patients.

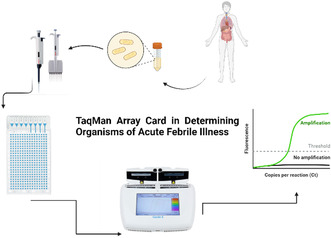

## 1 INTRODUCTION

Acute febrile illness (AFI) is characterized by a sudden onset of fever and other symptoms that are commonly caused by multiple pathogenic agents. Despite being a commonly presented symptom in healthcare settings, the disease burden of AFI is often underestimated and under‐reported.^1^, ^2^ The presence of various causative agents for AFI, along with its frequently occurring symptoms, and limited resources in tropical countries, creates difficulties in diagnosing, treating, and implementing public health responses for both endemic and epidemic diseases globally.^3^, ^4^ In Bangladesh, major causes of AFI include dengue, malaria, typhoid fever, leptospirosis, and rickettsial infection.^5^, ^6^, ^7^, ^8^, ^9^ The case fatality rate varies between 5% and 24% among hospitalized adults with AFI.^10^, ^11^

Identifying the specific infectious agent causing AFI is challenging due to the nonspecific clinical presentation, diverse causes, various specimen types, and the need for multiple laboratory techniques. Prompt diagnosis is crucial in preventing a negative outcome of the illness, as delayed appropriate antibiotic treatment can decrease survival chances by 7.6% per hour.^12^ The blood culture test is the current standard for detecting bacterial infection in patients with AFI. However, it has limitations in detecting slow‐growing, intracellular, and fastidious microorganisms and in patients who have received antibiotics.^13^, ^14^ Due to its poor sensitivity of about 60%, a blood culture test may cause patients to skip timely antibiotic doses.^15^, ^16^, ^17^ Nucleic acid amplification (NAA) based approaches might reduce the turnaround time, and multiplex polymerase chain reaction (PCR) could simultaneously detect multiple pathogens.^18^ Although PCR assays can detect multiple organisms in a single reaction through multiplexing, screening a large number of samples for several pathogens simultaneously can be extremely labor‐intensive and impractical. Therefore, the development of a rapid and efficient nucleic acid‐based molecular diagnostic method capable of identifying multiple microorganisms of AFI could greatly improve patient care. The TaqMan array card (TAC) is a multiplex real‐time PCR with 384 wells, based on microfluidics, and is especially suitable for samples of only 1/2 μL volume. It can analyze 24–384 pathogenic targets simultaneously and its closed system structure reduces the possibility of contamination.^19^

In Bangladesh, limited data are available regarding the etiology of AFI. As of today, few studies have been conducted using TAC technology to identify the causative agents of AFI in hospitalized patients. Therefore, our objective was to evaluate the effectiveness of TAC in detecting infectious bacteria, viruses, protozoa, and fungi in the blood samples of hospitalized AFI patients compared with blood cultures.

## 2 MATERIALS AND METHODS

### 2.1 Study design

The cross‐sectional study included 120 patients with an unknown etiology of fever who were admitted to the Department of Medicine at Chittagong Medical College Hospital (CMCH) and Bangladesh Institute of Tropical and Infectious Diseases (BITID) between January and December 2018. The participants or their guardians gave written consent after being informed of the study's purpose, and the research adhered to the ethical standards set by the Declaration of Helsinki. Before recording the patient's full history and conducting a related physical examination, their consent was obtained. The study was approved by the ethical review committee at Chittagong Medical College (Memo No. CMC/PG/2018/382).

### 2.2 Inclusion criteria

All patients attending the hospitals with a diagnosis of AFI were at first assessed for eligibility. Patients of either sex with documented fever (≥38°C/100.4°F) or on admission oral temperature (≥38°C/100.4°F) for 3–14 days and age above 12 years with unknown etiology of fever were enrolled in this study.

### 2.3 Excluding criteria

Patients who were under the age of 12 and had incomplete medical records during the study period were excluded.

### 2.4 Blood collection

Blood samples were collected from enrolled patients by a trained nurse by maintaining proper precautions and aseptic conditions. A total of 10 mL of venous blood were collected from each patient after physical examination for different assay purposes, and distributed accordingly: 5 mL in aerobic FAN bottle for automated BacT/Alert blood culture system, 1 mL in sodium heparin tube for typhoid/paratyphoid (TP) test, 2 mL in EDTA tube for TAC assay and 2 mL in EDTA tube for complete blood count (CBC). Following the blood collection, EDTA, or sodium heparin tubes were flipped 2–3 times for homogenous mixing. Blood in the EDTA tube for TAC assay was stored at −80°C.

### 2.5 Blood culture

Blood culture was performed in an automated BacT/Alert blood culture system. Briefly, 5 mL of blood samples were collected from each patient and immediately inoculated into aerobic FAN blood culture bottles. These bottles were then inserted into BacT/Alert incubator unit for 5/6 days. A positive or negative FAN bottle for bacterial growth was subjected to subculture on MacConkey (MC) agar, chocolate agar, and blood agar (which contained 5% sheep blood) for 2 days. The MC agar plates were then aerobically incubated at 35°C, while the chocolate and blood agar plates were incubated in microaerophilic growth conditions (5% CO_2_ and 35°C). Microbial isolates were identified using the Clinical and Laboratory Standards Institute (CLSI) guidelines.^20^

### 2.6 TPTest for detecting *Salmonella* Typhi/Paratyphi

Besides the automated BacT/Alert blood culture system, TPTest was also performed for identifying *Salmonella*‐specific IgA responses in the lymphocyte culture supernatant as previously described.^21^ In summary, unstimulated peripheral blood mononuclear cells (PBMCs) were cultured at a concentration of 10^7^ cells/mL in RPMI 1640 medium, with heat‐inactivated fetal bovine serum (10%), streptomycin (100 μg/mL), penicillin (100 U/mL), pyruvate (100 mM), and L‐glutamine (200 mM). The culture was incubated at 37°C with 5% CO_2_ for several time periods. Following this, the culture supernatants were collected and analyzed for IgA antibodies that were specific to *Salmonella* Typhi membrane preparation (MP) using the enzyme‐linked immunosorbent assay (ELISA) method.

### 2.7 Total nucleic acid extraction from blood samples for TAC assay

Total nucleic acid is extracted from blood specimens using the High Pure Viral Nucleic Acid Large Volume Kit (Roche). Briefly, 200 μL freshly prepared working solution containing carrier RNA was added into 200 μL blood samples. Fifty microliters of Proteinase K solution was mixed with the solution. The solution was incubated at (+72 ± 2) °C for 10 min. After adding 100 μL of binding buffer and thorough mixing, the solution was transferred to the High Pure Filter Tube, inserted into a collection tube, and centrifuged at 8000 *g* for 1 min. The collection tube was discarded with flow‐through and the High Pure Filter Tube was then inserted into a fresh collection tube. Five hundred microliters of inhibitor removal buffer was added to it and centrifuged at 8000 *g* for 1 min. The High Pure Filter Tube was placed into another collection tube and 450 μL of washing buffer was added and centrifuged the tube at 8000 *g* for 1 min. Only the flow‐through was discarded and repeated the washing step. The isolated nucleic acid was eluted from the filter by adding 50 μL elution buffer and was collected in a 1.5 mL microcentrifuge tube for storing at −80°C for the TAC assay. Extrinsic controls PhHV (Phocine Herpesvirus) and bacteriophage MS2 were introduced to each sample to measure extraction and amplification effectiveness during lysate preparation. During the extraction of nucleic acid, an experimental blank was maintained throughout the protocol and later assayed to check out contamination.

### 2.8 Identifying bloodstream pathogens by TAC assay

Nucleic acid extract was mixed with TaqMan Fast Virus 1‐Step Master Mix (Applied Biosystems) in a 100 μL reaction mixture and then pipette the mixture into the inlet port of each channel. The card was centrifuged twice for 1 min at 97 *g* to fill the well, sealed the card to close the well, and the inlet ports were removed. Within 10 min card was ready to run on the QuantStudio™ 7 Flex instrument (Applied Biosystems, Thermo Fisher Scientific). Primers and probes targeting AFI‐associated microorganisms developed by Liu et al.,^22^ were used in this study (Table S1). The TAC was run using PCR cycling conditions comprising 10 min at 50°C and 20 s at 95°C followed by 45 two‐step cycles of 3 s at 95°C and 30 s at 60°C. A universal bacterial 16S assay was also included on the card for inferring the presence of bacterial pathogens that were not interrogated on the card. After 1.5 h the results were ready for analysis. The amplification data were analyzed by the conventional cycle‐threshold (*C*_t_) method. A *C*_t_ threshold value of 40 cycles was employed to distinguish between positive and negative samples for both clinical specimens and the negative control on the TAC.

### 2.9 Data analysis

Data were analyzed using statistical software (Statistical Package for Social Science, SPSS Version 23.0, IBM Corporation). Continuous variables were given as means ± SD, whereas categorical variables were presented as frequency and percentages. Sensitivity, specificity, positive predictive value, negative predictive value, and diagnostic accuracy for each test were computed, as were 95% confidence intervals. The gold standard for analytical comparison was blood culture. Statistical significance was defined as *p* < 0.05 and a confidence interval was set at 95%.

## 3 RESULTS

### 3.1 Characteristics of study participants

A total of 120 hospitalized AFI patients [70 (58.3%) from CMCH and 50 (41.7%) from BITID] were enrolled in the study for evaluating the role of the TAC test in diagnosing hospitalized AFI patients.

The calculated mean age of the patients was 31.88 (±14.9) years with male predominance (57.5%) and a majority of them were from rural areas. The most prevalent symptom other than fever was a headache in 67 patients followed by nausea, vomiting, headache, back pain, abdominal pain, rash, cough, red eye, retro‐orbital pain, etc. Anemia was the commonest sign followed by dehydration, red eye, jaundice, conjunctival hemorrhage, skin rash, cervical lymphadenopathy, hepatosplenomegaly, etc.

Characteristics of the fever of the AFI patients showed that the mean temperature on admission was 102.04 ± 1.21°F. In most cases, temperature was high. The average duration of fever was 9 days, which indicates patients are suffering from fever for a long duration without specific treatment. The majority of the patients had a history of receiving both oral and injectable antibiotics before blood sample collection (Table 1).

**TABLE 1:** Description of the fever of the patients (*n* = 120).

Fever	
Duration (days)	
Mean ± SD	9.34 ± 3.49
Range	3–14
Admission temperature (°F)	
Mean ± SD	102.04 ± 1.21
Range	100–105
Presample antibiotic, *n* (%)	
Yes	109 (90.8)
No	11 (9.2)

### 3.2 The positivity rate of different tests among patients with AFI

To determine the etiology of AFI TAC, blood culture and TP test were done. Among these three tests, TAC shows the highest sensitivity followed by the TP test and blood culture. All groups of organisms of AFI such as bacteria, viruses, fungi, and protozoa were identified by TAC assay and the major cause was bacteria. Out of 120 AFI cases, at least 1 pathogen was identified in 40% (48) specimens by TAC test. In blood culture 7.5% (9) specimens were positive. The commonest culture‐positive organism is *Salmonella*, followed by *Acinetobacter* and *Escherichia coli*. TP test was found positive in 24.16% (29) specimens (Table 2).

**TABLE 2:** Frequency of positivity rate of different tests in AFI patients (*n* = 120).

Test	Organism type	Frequency (%)
TAC positive	Bacteria	34 (70.8)
	Virus	9 (18.8)
	Fungus	2 (4.2)
	Virus + Protozoa	2 (4.2)
	Bacteria + Virus	1 (2.1)
Total		48 (40)
Blood culture	*Salmonella* Typhi	6 (66.7)
	Acinetobacter	2 (22.2)
	*Escherichia coli*	1 (11.1)
Total		9 (7.5)
TPTest positive	*Salmonella* Typhi	29 (100)
Total		29 (24.16)

### 3.3 Detection of co‐infection using TAC

Of 120 cases, in 48 cases either single or multiple organisms were detected. Besides, the detection of one organism, in eight cases co‐detection of more than one organism was observed. Two pathogens were detected in 5% (6). Three and four pathogens were detected in 0.8% (01) specimens. The most common co‐pathogen was dengue followed by *Salmonella* and *Rickettsia* (Table 3).

**TABLE 3:** Detection of single and multiple organisms by TAC assay.

Detection frequency	Frequency (*n*)	Percentage
No organism detected	72	60.0
One organism detected	40	33.4
Co‐detection of one organism	6	5.0
Co‐detection of two organism	1	0.8
Co‐detection of three organisms	1	0.8

### 3.4 Pathogen‐specific attributable frequency of microorganisms using TAC quantitative molecular diagnostics

**FIGURE 1:**
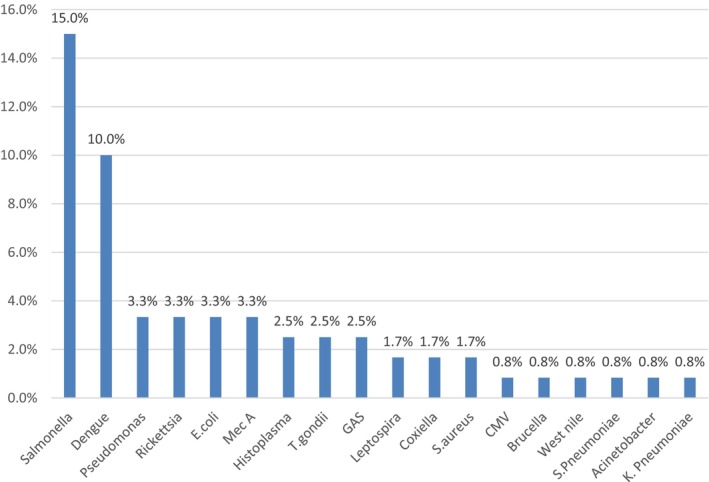
Organisms found in TAC assay.

TAC assay identified 18 different pathogens including 13 bacteria, three viruses, one fungus, and one protozoa. The most prevalent bacteria *Salmonella* Typhi was recovered from 15% of samples and the leading virus was Dengue (10%). Toxoplasma is the only protozoa found in 2.5% of cases. Other pathogens were *Ricketssia* (3.3%), *Pseudomonas* (3.3%), *E. coli* (3.3%), *Leptospira* (1.7%), *Coxiella* (1.7%), *Histoplasma* (2.5%), *Toxoplasma* (2.5%), and Cytomegalovirus (0.8%) (Figure 1).

### 3.5 The sensitivity and specificity of TAC compared to blood culture

Among 120 samples TAC test was positive for bacteria in 14 samples and blood culture was positive in only nine samples (Table 4). Of these nine positive culture‐positive cases, four cases were TAC‐positive revealing a sensitivity of the TAC test of 44.4% considering blood culture as the gold standard. The specificity of the TAC test and overall accuracy of the TAC test in comparison to blood culture was, respectively, 90.99% and 87.50%. PPV was 28.57% which means the probability that the culture is present when the TAC test is positive. NPV was 95.28% indicating the probability that culture is negative when the TAC test is negative.

**TABLE 4:** Diagnostic accuracy of TAC compared to blood culture (*n* = 120).

	Blood culture		Total
	Positive	Negative	
TAC test			
Positive	4	10	14
Negative	5	101	116
Total	9	111	120

Statistic	Value	95% CI
Sensitivity	44.44%	13.70%–78.80%
Specificity	90.99%	84.06%–95.59%
Positive predictive value	28.57%	13.52%–50.59%
Negative predictive value	95.28%	91.82%–97.32%
Accuracy	87.50%	80.22%–92.83%

### 3.6 Diagnosis of enteric fever using TP test

TP Test was positive in 29 samples out of 120 and blood culture was positive in only six samples (Table 5). All of the culture‐positive cases were also TP‐positive revealing a sensitivity of TP test 100% considered blood culture as the gold standard. The specificity and overall accuracy of the TP test in comparison to blood culture were, respectively, 79.82% and 80.83%.

**TABLE 5:** Diagnostic accuracy of TP test compared to blood culture (*n* = 120).

	Blood culture		Total
	Positive	Negative	
TP test			
Positive	6	23	29
Negative	0	91	91
Total	6	114	120

Parameter	Value (%)	95% CI
Sensitivity	100	54.07%–100%
Specificity	79.82	71.28%–86.76%
PPV	20.69	15.33%–27.32%
NPV	100	
Accuracy	80.83	72.64%–87.44%

## 4 DISCUSSION

This study provides us with a valuable and informative diagnostic assessment of AFI cases in tertiary hospitals. Of 120 enrolled patients, at least one organism was identified in 48 (40%) cases by TAC assay.

Bacteria, viruses, protozoa, and fungi all types of organisms were detected by TAC assay from AFI patients. The most prevalent bacteria was *Salmonella* Typhi (15%) and the virus was *Dengue* (10%). We also found *Rickettsia*, *E. coli*, *Pseudomonas*, *Leptospira*, *Coxiella*, *Histoplasma*, and *Toxoplasma*. A study from Tanzania^23^ showed that *Plasmodium* was the most common organism among the total 56 pathogens. Other organisms were *Leptospira* in 3% of cases and *Salmonella enterica*, *Rickettsia, Bartonella*, *Coxiella burnetii*, and *West Nile* virus in 1% of cases. Normally, immediately after admission, RDT was done for most of the AFI patients hailing from the hilly area of Chattogram by the hospital/medicine department. To meet the inclusion criteria, we had to exclude RDT‐positive cases. Hence, we did not get any plasmodium in our study.

After analysis of amplification curves of TAC‐positive cases, we found some uncommon organisms like *West Nile*, *Histoplasma*, *Coxiella*, *Brucella*, *Toxoplasma*, and *Leptospira* as the cause of AFI. The history and examination findings of these cases were clinically relevant. A study in six tertiary hospital‐based febrile patients of Bangladesh identified *Rickettsia* (263), *Coxiella* (10), *Leptospira* (2), and *Bartonella* (1) among 720 patients by serological test.^6^ The findings of our study were closely reflected in their study.

We found only one case of West Nile virus, though this arbovirus is uncommon in our country. This virus was first time detected in Bangladesh. As the sample collection covered monsoon and post‐monsoon season so, dengue was the second‐highest organism found in this study. But we did not find any Chikungunya virus. Previous literature suggested that for the majority of arboviruses, viremia tends to peak prior to the onset of clinical illness, thus our acute sampling time frame (3–14 days post‐symptom onset) might have not been optimized to detect such bloodstream agents.^19^

We validated TAC finding only for bacterial pathogens by blood culture test. However, only three bacterial species *Salmonella* Typhi, *Acinetobacter*, and *E. coli* were identified by nine positive culture results. The sensitivity and specificity of the TAC assay for bacterial pathogens in comparison to blood culture samples were 44.44% and 90.99%, respectively. We anticipated that the sensitivity of PCR for bacterial pathogens on a single blood specimen, particularly with TAC and its small sample volume may be mediocre versus that of culture. A meta‐analysis of commercial PCR tests for bacterial detection in samples of sepsis patients showed a sensitivity of only 61%–80% versus blood culture.^24^ This finding is specifically well known for *Salmonella* Typhi. In another study, the sensitivity of PCR in blood was 50% versus blood culture.^25^ In a study for AFI outbreak investigation and surveillance TAC was 71% sensitive versus that of blood culture.^18^ Our study is in line with these observations. However, organism‐specific serological tests, ELISA, or individual real‐time PCR were not performed for any AFI patient in our study.

We have co‐detected up to three organisms. Co‐detection of one organism was found in six cases but two and three organisms in one case each. These results of co‐detection are consistent with another AFI study^23^ where no organism was found in 50% of cases and two organisms in 0.8% of cases. Further studies are needed to understand the clinical diagnosis and outcome of patients who were PCR‐positive for multiple pathogens.

Of the 120 specimens, five were culture‐positive/TAC‐negative. The physical condition of sample collection, transportation to the laboratory, and storage duration of the whole blood samples could greatly affect the quantity and quality of nucleic acids (DNA, RNA),^25^, ^26^ which in turn, further contribute to the failure of TAC assay. During our study, blood samples were collected from CMCH and BITID and transported to the laboratory with the appropriate carrier condition. All the blood samples were extracted and stored for 7–15 days at BITID and then the extracted nucleic acids were transported to icddr,b for TAC analysis. These five samples were collected after antibiotic treatment. Thus, the quality of nucleic acid might fall during those mentioned steps.

Although the patients presented with clinical symptoms of infection but 111 samples showed negative results in the blood culture test. Among these 111, bacteria were identified in 28 specimens by the TAC assay. In previous studies,^18^ to verify this type of discrepancy, TAC results were verified by PCR/sequencing and found that the results were the same as those of TAC but we could not confirm these organisms by individual PCR/sequencing. Most of our cases received several doses of antibiotics before blood collection. As a result, bacterial load was low in the blood. So, the findings were not the same as we thought.

According to our study, bacterial pathogens, specifically, *Salmonella*, *Pseudomonas*, *Rickettsia*, and *E. coli*, were predominant among hospitalized AFI patients. Our findings were consistent with the previous study by Ahmed et al.,^27^ in which they found a 13.6% positive scenario in the BACT/Alert blood culture system. Among the positive cases, gram‐negative bacteria accounted for 72.1%, with *Salmonella Typhi* being the most abundant isolated microorganism (36.9%).

The culture positivity rate in the present study was low. The variations in culture positivity rate were related to different factors such as the number of blood cultures, the volume of the blood taken, the type of the culture broth used, the patient's selection criteria, using a clinical decision‐making rule to select the clinically suspicious patients of bacteremia, different socioeconomic background, etc.

We found 29 were TP positive and blood culture for *Salmonella* was positive in only six cases. The sensitivity of the TP test was 100% and the specificity was 79.82% in comparison to the blood culture. The findings of our study were in accordance with the findings observed in another study done in Dhaka, Bangladesh.^28^

Our analysis detected a wide range of organisms among 120 AFI patients. Other than common organisms some different organisms were detected for which tests are usually not done in our routine practice. Investigations for these organisms are time‐consuming and costly. The sensitivity of the TAC assay for bacterial pathogens was 44.4% which was in line with other studies. The negative predictive value was high and the probability of disease could be excluded.

The study has several limitations. First we compared only the TAC‐positive bacteria with culture but we could not perform individual real‐time PCR for all groups of organisms due to a lack of resources. Second, the sample size was limited due to less availability of the TAC. Also, we were not able to cover all seasons as the sample was collected from February to October. So the etiological variation of AFI in the different seasons could not be evaluated.

In many low‐ and middle‐income countries (LMICs), diagnostic capacity remains limited, and the etiologies in AFI are poorly documented which impedes the ability to develop effective clinical algorithms or to make informed clinical decisions. Thus, the need to develop diagnostics for enhanced surveillance in LMICs and to rapidly and comprehensively assess etiology. The multiplex TAC assay was developed to avoid contamination and improve the detection efficiency for multiple pathogens within a short time. Moreover, the assay panels can be easily customized according to the target microorganisms being investigated. The use of TAC assay has been studied in several outbreak situations in identifying the etiology of diseases in many LIMCs.^22^, ^29^, ^30^ In our study, we performed the TAC assay in admitted AFI populations from two hospitals in the Chittagong region, where the diagnosis of AFI was limited due to available laboratory tests. More studies in broader target populations and in different research settings, disease surveillance, or investigating outbreaks of unknown etiology are needed to justify the use of TAC assay in the rapid and simultaneous detection of multiple pathogens over other tedious and expensive molecular or conventional culture tests.

## 5 CONCLUSION

TAC assay is useful for the detection of multiple organisms of AFI. It provides us a diversity of previously undetected pathogens of AFI which was missed by blood culture. We documented that this test was highly specific so, there was no false‐positive result. We may use this newer molecular technique as a diagnostic tool for better characterization and early diagnosis of multiple contributing agents of AFI. Greater awareness will be developed among clinicians about etiology of AFI which will help in early diagnosis and management of AFI.

## CONFLICT OF INTEREST STATEMENT

The authors declare that they have no known competing financial interests or personal relationships that could have appeared to influence the work reported in this article.

## Supporting information


Table S1

## Data Availability

Data can be shared based on the reader's reasonable request and priority base and some restrictions will apply.
