# Supplementary material for: Role of TaqMan array card in determining causative organisms of acute febrile illness in hospitalized patients
Source: J Clin Lab Anal. 2023 Jul 27;37(13-14):e24948. doi: 10.1002/jcla.24948 (PMC10492456; doi:10.1002/jcla.24948)
Supplement: Supplementary file 1 — Table S1 [file JCLA-37-e24948-s001.docx]

Supplementary Table 1: Primer and probe sequences for the TaqMan array card assays

| Pathogens | Target | Sequence (5′–3′) |
| --- | --- | --- |
| **Virus** | | |
| Chikungunya | NSP4 | F: TCACTCCCTGYTGGACTTGATAGA |
|  |  | R: TTGACGAACAGAGTTAGGAACATACC |
| Dengue | 3′NC | F: GGATAGACCAGAGATCCTGCTGT |
|  |  | R: CATTCCATTTTCTGGCGTTC |
|  |  | R: CAATCCATCTTGCGGCGCTC |
| Nipah | NP | F: CTGGTCTCTGCAGTTATCACCATCGA |
|  |  | R: ACGTACTTAGCCCATCTTCTAGTTTCA |
| West Nile | 3’ NC | F: CAGACCACGCTACGGCG |
|  |  | R: CTAGGGCCGCGTGGG |
| **Protozoa** | | |
| *Plasmodium* spp. | 18S | F: GCTCTTTCTTGATTTCTTGGATG |
|  |  | R: AGCAGGTTAAGATCTCGTTCG |
| *Leishmania* spp. | 18S | F: AAGTGCTTTCCCATCGCAACT |
|  |  | R: GACGCACTAAACCCCTCAA |
| *Trypanosoma brucei* | 18S | F: CGCCAAGCTAATACATGAACCAA |
|  |  | R: TAATTTCATTCATTCGCTGGACG |
| **Fungus** | | |
| *Cryptococcus neoformans* | *5.8S and ITS* | F:CCTGTTGGACTTGGATTTGG |
|  |  | R: AGCAAGCCGAAGACTACC |
| *Histoplasma capsulatum* | *5.8S and ITS* | F:GTCTGAGCATGAGAGCGATAATAATC |
|  |  | R: CGCTGCGTTCTTCATCGA |
| **Bacteria** | | |
| *Salmonella* Typhi | STY0201 | F: CGCGAAGTCAGAGTCGACATAG |
|  |  | R: AAGACCTCAACGCCGATCAC |
| *Leptospira* spp. | LipL32 | F: CCCTAIGGATCTGTRATCAACTA |
|  |  | R: GAACTCCCATTTCAGCGATT |
| *Rickettsia* spp. | 23S | F: AGCTTGCTTTTGGATCATTTGG |
|  |  | R: TTCCTTGCCTTTTCATACATCTAGT |
| *Brucella* spp. | IS711 | F: GCTTGAAGCTTGCGGACAGT |
|  |  | R: GGCCTACCGCTGCGAAT |
| *Coxiella burnetii* | IS1111 | F: CCGATCATTTGGGCGCT |
|  |  | R: CGGCGGTGTTTAGGC |
| *Bartonella* spp. | ssrA | F: GGCTAAATIAGTAGTTGCAAAYGACA |
|  |  | R: GCTTCTGTTGCCAGGTG |
| *S. pneumoniae* | *lytA* | F: ACGCAATCTAGCAGATGAAGCA |
|  |  | R: TCGTGCGTTTTAATTCCAGCT |
| *M. tuberculosis* | *orfB* | F: GGCTGTGGGTAGCAGACC |
|  |  | R: CGGGTCCAGATGGCTTG |
| *H. influenzae* | *bexA* | F: GGACAAACATCACAAGCGGTTA |
|  |  | R: TGCGGTAGTGTTAGAAAATGGTATTATG |
| *Escherichia coli* | *uidA* | F:GAGCATCAGGGTGGCTATACG |
|  |  | R:ATAGTCTGCCAGTTCAGTTC |
| *Klebsiella pneumoniae* | *Diguanylate cyclase* | F:TGCAGATAATTCACGCCCAG |
|  |  | R: ACCCGCTGGACGCCAT |
| *Listeria monocytogenes* | *hly* | F:TTTCATCCATGGCACCACC |
|  |  | R:ATCCGCGTGTTTCTTTTCGA |
| *Acinetobacter baumannii* | *ompA* | F:AGTTCTTGGTGGTCACTTGAAGC |
|  |  | R:TTAACTCTTGTGGTTGTGGAGCA |
| *Pseudomonas aeruginosa* | *23srRNA* | F:TCCAAGTTTAAGGTGGTAGGCTG |

|  |  | R: ACCACTTCGTCATCTAAAAGACGAC |
| --- | --- | --- |
| *N. meningitidis* | *ctrA* | F:GCTGCGGTAGGTGGTTCAA |
|  |  | R: TTGTCGCGGATTTGCAACTA |
| *K. oxytoca* | *bla* | F: ATGCGCTGGGCGAACA |
|  |  | R: CTGCTGCGGCTGGGTAA |
| *E. faecalis* | *recN* | F: GTATCGCGCACTCGAAGCC |
|  |  | R: CATGTCCATTCTTTGGGCAA |
| *S. pyogenes* | *spy* | F: GCACTCGCTACTATTTCTTACCTCAA |
|  |  | R: GTCACAATGTCTTGGAAACCAGTAAT |
